# Exploiting signaling rewiring in cancer cells with co‐existing oncogenic drivers

**DOI:** 10.1002/1878-0261.13547

**Published:** 2023-10-31

**Authors:** Roberto Chiarle, Chiara Ambrogio

**Affiliations:** ^1^ Department of Pathology Boston Children's Hospital and Harvard Medical School Boston MA USA; ^2^ Department of Molecular Biotechnology and Health Sciences, Molecular Biotechnology Center University of Torino Torino Italy

**Keywords:** driver oncogenes, drug resistance, signaling rewiring

## Abstract

The development of tailored therapies designed to specifically target driver oncogenes has initiated a revolutionary era in cancer biology. The availability of a growing number of selective inhibitors has generated novel experimental and clinical paradigms. These represent an opportunity and a challenge for researchers and clinicians to delve deeper into the intricate dynamics of cancer development and response to treatment. By directly inhibiting key driver oncogenes involved in tumor initiation and progression, scientists have an unprecedented opportunity to conduct longitudinal and clonal evolutionary studies of how cancer cells adapt, rewire, and exploit conflictive or overlapping signaling dependencies in response to treatment *in vitro* and *in vivo*. This challenge has to be progressively resolved to discover more effective and personalized cancer therapies.

## Graphical abstract

Personalized cancer therapies mark a groundbreaking era in cancer research. The chance to identify new vulnerabilities stemming from cancer cell adaptation, signaling rewiring, and treatment response is now a reality. In this battle with tumors, researchers and clinicians must carefully consider their moves, just like chess players, to anticipate the response of tumors and strategize the most precise and effective therapy.

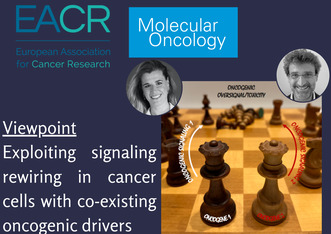

**Abbreviations**
ALK: anaplastic lymphoma kinase
EGFR: epidermal growth factor receptor
KRAS: Kirsten rat sarcoma virus oncogene homolog
NTRK: neurotrophic tyrosine receptor kinase
RET: rearranged during transfection proto‐oncogene

In cancer biology, the concept of “oncogenic sweet spot” refers to the optimal signaling input resulting in best cell fitness output [1]. This optimal input is the result of genomic information connected to transcriptional regulation and post‐translational modifications. In this context, recurrent patterns of mutations that are either co‐occurring or mutually exclusive in human cancers have been progressively identified largely due to the exponentially growing amount of data provided by improved sequencing technology [2, 3]. These stereotyped patterns tend to show the mutual exclusivity of key oncogenic drivers that can orchestrate a full program of cell transformation. Experimental models have demonstrated that the co‐occurrence of conflictive oncogenes is not favored due to the cellular stress that derives from ‘over‐signaling’. This would result in a reduced fitness for tumor cells that are forced to express two mutually exclusive oncogenes [4, 5] or forced to overexpress individual oncogenes [6, 7].

Current knowledge regarding drug resistance in cancer often revolves around the identification of specific genetic mutations responsible for stable, inheritable resistance states. However, there is growing recognition of adaptive and potentially reversible mechanisms of drug resistance. These plastic mechanisms represent dynamic phenotypic diversity present within tumor cell populations which is dependent on individual oncogenic sweet‐spot, suggesting that disrupting these states could be highly advantageous. Therapeutic efforts can be focused on shifting the drug‐resistant subpopulation toward a state that represents the drug sensitivity of the majority of cells, promoting greater uniformity among tumor cell subpopulations.

Systematic sequencing of tumor biopsies at relapse or longitudinal sampling of circulating tumor DNA in clinical trials and real‐world patients have yielded vast amounts of data that have substantially broadened the understanding of the genetic basis of tumor cell adaptation to the blockade of driver oncogenic signaling. Typically, a sustained inhibition of driver oncogenes (i.e., EGFR, ALK, KRAS, RET, NTRK) shapes the selection of clonal populations that are driven by co‐existing oncogenes that never occur in treatment‐naïve tumors [8, 9, 10, 11]. Previous studies have suggested that the oncogenic sweet‐spot for a positive balance between proliferation and apoptosis can be found by tumor cells in multiple ways. One example being a single driver truncating mutation in treatment‐naïve tumors or the co‐existence of two or three driver oncogenes when targeted therapy downregulates the oncogenic signaling and selects for newly occurring mutations.

While our understanding of these mechanisms is still incomplete, an immediate question that arises is whether this tumor adaptability can be exploited as a liability for cancer treatment when two potent and druggable oncogenes co‐exist. In some models, preclinical data have demonstrated that the co‐existence of potent driver oncogenes is detrimental for cell fitness in untreated tumor cells. In contrast, this co‐existence is selected upon oncogenic driver inhibition in experimental models and patients (Fig. 1A) [12]. Would drug withdrawal in this context result in anti‐tumor activity due to a combined oncogene toxicity? If so, what would be the best strategy to exploit this co‐existence of multiple driver oncogenes?

**Fig. 1:**
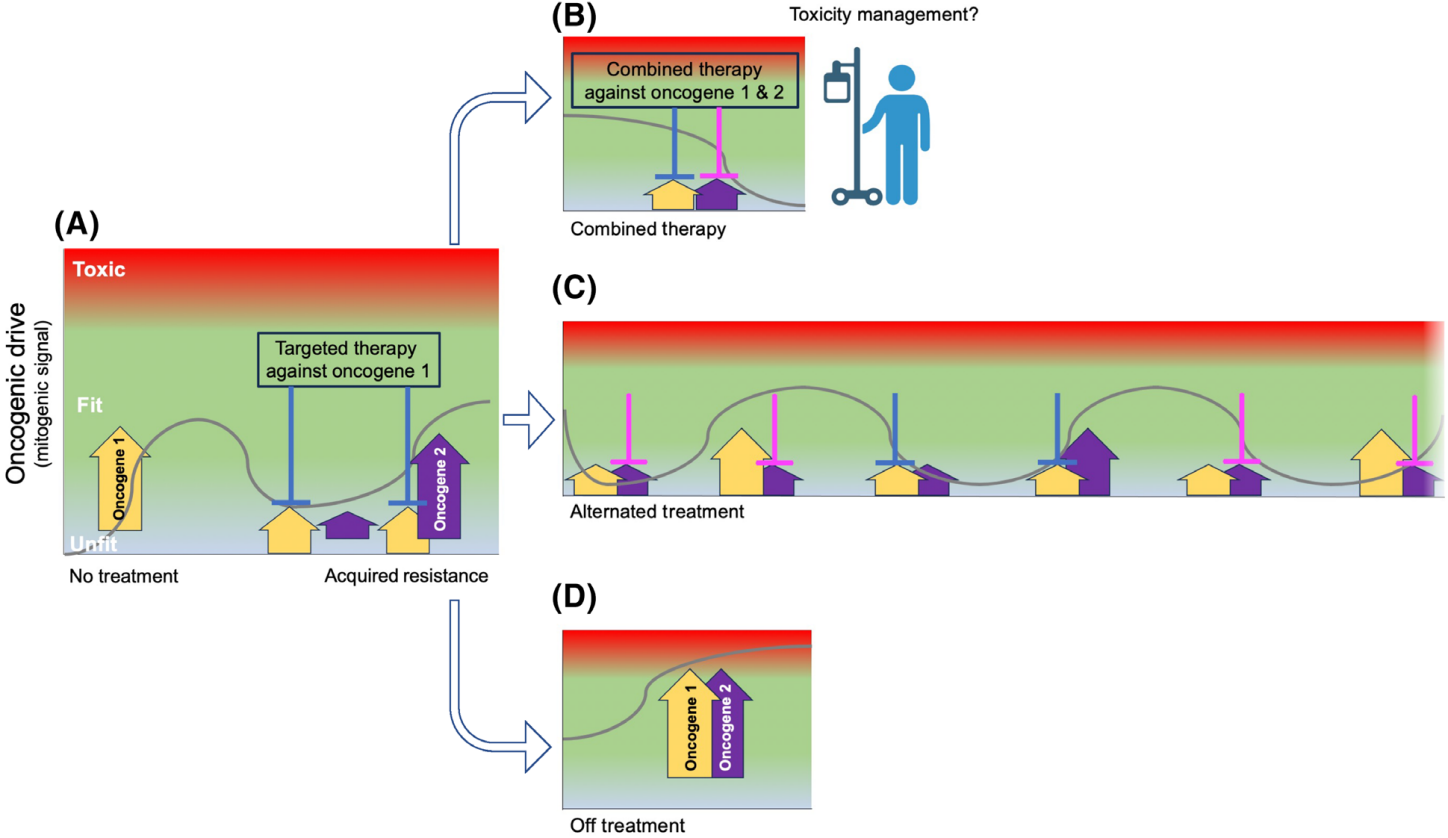
Targeted treatment allows for acquisition of secondary oncogenic alterations otherwise incompatible in untreated tumors due to excessive signaling (A). Potential alternative treatments in the presence of co‐existing oncogenic drivers (B–D).

One immediate approach would be a dual therapy that simultaneously targets both mutated driver oncogenes (Fig. 1B). While reasonable in principle, it is not infrequent to observe non‐tolerable toxicities in patients with drug combinations. The type and severity of these toxicities cannot be fully predicted and depend on each drug combination together with the individual patient's response. If toxicities are observed, alternative approaches can be envisioned. One possibility is a monotherapy that sequentially targets each of the co‐existing driver oncogenes (Fig. 1C). The key element of successful sequential drug treatment strategies relies on the induction of a susceptible state by the initial therapy targeting the truncal driver oncogene and forcing cancer cells to rewire to a new oncogene dependency.

This approach would likely alleviate the toxicity of combined therapies and could be achieved by designing treatment regimens that exhibit synergy without administering both drugs concurrently [12]. Such a feat could be accomplished if the initial drug provokes a considerable vulnerability in cancer cells, which must persist beyond the interruption of the first treatment and can be exploited by a second drug to eradicate remaining vulnerable cells [13]. An additional provocative strategy could be the sudden withdrawal of any treatment. While this approach could be a gamble, the abrupt over‐signaling induced by the co‐existing oncogenes should bring the cells over the threshold of optimal fitness and induce cell‐cycle arrest or apoptosis as a reaction to the rapid and potent unleashing of oncogenic stress (Fig. 1D). Whether one of these approaches would be best for each patient remains to be determined by clinicians. In this context, it is expected that each of these approaches could have a different efficacy based on the tumor subtype, the features of the co‐existing mutations, and the potency and safety profiles of the drugs available.

In all these treatment scenarios, the goal is to delay or prevent the emergence of drug resistance through sequential administration of targeted therapies, offering a treatment schedule to mitigate possible constraints on dosage due to cumulative toxicities between the combined agents. In other words, a complete eradication of tumor cells might be difficult to achieve with multiple drug combinations due to the barrier imposed by toxicity. However, there could possibly be alternative ways to exploit the growing arsenal of targeted therapies to prolong the fight with drug‐resistance cancer cells while improving the quality of life of cancer patients.

## Conflict of interest

CA received research fees from Revolution Medicines, Verastem, Roche and Boehringer‐Ingelheim. RC is the scientific founder of ALKemist Bio.

## Author contributions

RC and CA conceived and wrote the paper.

## References

[1] Li S , Balmain A , Counter CM . A model for RAS mutation patterns in cancers: finding the sweet spot. Nat Rev Cancer. 2018;18:767–777. 30420765 10.1038/s41568-018-0076-6

[2] El Tekle G , Bernasocchi T , Unni AM , Bertoni F , Rossi D , Rubin MA , et al. Co‐occurrence and mutual exclusivity: what cross‐cancer mutation patterns can tell us. Trends Cancer. 2021;7:823–836. 34031014 10.1016/j.trecan.2021.04.009

[3] Gainor JF , Varghese AM , Ou SHI , Kabraji S , Awad MM , Katayama R , et al. ALK rearrangements are mutually exclusive with mutations in EGFR or KRAS: an analysis of 1,683 patients with non‐small cell lung cancer. Clin Cancer Res. 2013;19:4273–4281. 23729361 10.1158/1078-0432.CCR-13-0318 PMC3874127

[4] Ambrogio C , Barbacid M , Santamaría D . In vivo oncogenic conflict triggered by co‐existing KRAS and EGFR activating mutations in lung adenocarcinoma. Oncogene. 2017;36:2309–2318. 27775074 10.1038/onc.2016.385

[5] Unni AM , Lockwood WW , Zejnullahu K , Lee‐Lin S‐Q , Varmus HE . Evidence that synthetic lethality underlies the mutual exclusivity of oncogenic KRAS and EGFR mutations in lung adenocarcinoma. Elife. 2015;4:e06907. 26047463 10.7554/eLife.06907 PMC4478584

[6] Chang L , Jung N , Atari A , Rodriguez D , Kesar D , Song T , et al. Systematic profiling of conditional pathway activation identifies context‐dependent synthetic lethalities. Nat Genet. 2023;55:1709–1720. 37749246 10.1038/s41588-023-01515-7

[7] Ceccon M , Merlo MEB , Mologni L , Poggio T , Varesio LM , Menotti M , et al. Excess of NPM‐ALK oncogenic signaling promotes cellular apoptosis and drug dependency. Oncogene. 2016;35:3854–3865. 26657151 10.1038/onc.2015.456 PMC4907875

[8] Diaz LA , Williams RT , Wu J , Kinde I , Hecht JR , Berlin J , et al. The molecular evolution of acquired resistance to targeted EGFR blockade in colorectal cancers. Nature. 2012;486:537–540. 22722843 10.1038/nature11219 PMC3436069

[9] Awad MM , Liu S , Rybkin II , Arbour KC , Dilly J , Zhu VW , et al. Acquired resistance to KRASG12C inhibition in cancer. N Engl J Med. 2021;384:2382–2393. 34161704 10.1056/NEJMoa2105281 PMC8864540

[10] Reuss JE , Gandhi N , Walker P , Nieva JJ , Bustamante Alvarez JG , Carlisle JW , et al. Spectrum of acquired KRAS mutations in driver mutation‐positive non‐small cell lung cancer. J Clin Oncol. 2023;41:9069.

[11] Hua G , Zhang X , Zhang M , Wang Q , Chen X , Yu R , et al. Real‐world circulating tumor DNA analysis depicts resistance mechanism and clonal evolution in ALK inhibitor‐treated lung adenocarcinoma patients. ESMO Open. 2022;7:100337. 35123209 10.1016/j.esmoop.2021.100337 PMC8818928

[12] Settleman J , Fernandes Neto JM , Bernards R . Thinking differently about cancer treatment regimens. Cancer Discov. 2021;11:1016–1023. 33648929 10.1158/2159-8290.CD-20-1187

[13] Eser PÖ , Paranal RM , Son J , Ivanova E , Kuang Y , Haikala HM , et al. Oncogenic switch and single‐agent MET inhibitor sensitivity in a subset of EGFR‐mutant lung cancer. Sci Transl Med. 2021;13:eabb3738. 34516823 10.1126/scitranslmed.abb3738 PMC8627689

